# Biomarkers of stress across biological systems: conceptual and measurement perspectives

**DOI:** 10.3389/fpsyg.2026.1895140

**Published:** 2026-10-29

**Authors:** Sandra J. Weiss, Shaimaa Elrefaay, Victoria Keeton, Kayla Longoria, Harry Adynski, Cherry Leung

**Affiliations:** 1 University of California, San Francisco, San Francisco, CA, United States; 2 University of California, Davis, Davis, CA, United States; 3 The University of Texas Health Science Center at Houston, Houston, TX, United States

**Keywords:** biomarker, measurement, psychobiology, stress, stress reactivity

## Abstract

Stress and the ability to regulate stress substantially influence an individual's psychological health and wellbeing. There is growing evidence that the biological component of stress makes a unique contribution to understanding stress, yielding insights that are distinct from those specific to stress-related feelings and thoughts. The purpose of this paper is to provide an overview of selected biomarkers of stress as well as key issues regarding their use. Biomarkers associated with the hypothalamic–pituitary–adrenal axis (cortisol, dehydroepiandrosterone), the autonomic nervous system (heart rate variability, alpha-amylase), the immune system (cytokines and C-reactive protein), genetics (telomeres, DNA methylation), and multi-omics are discussed. Each biomarker is defined, along with the empirical and/or conceptual rationale for its use as a stress measure and examples of its research application. Collection, storage, and processing considerations for biospecimens or sensor-derived, physiologic data are addressed. For each biomarker, its utility in measurement of acute vs. chronic stress is differentiated, and established relationships to resilience are noted. In addition, key advantages and limitations of each marker are summarized. Lastly, significant areas for future research are highlighted that can move the field forward. Biomarkers represent a critical asset in the overall scope of research on stress and stress regulation, advancing knowledge of their health-related impact in fundamental and unique ways.

## Introduction

The experience of stress is ubiquitous in everyday life, from daily difficulties that erode health and wellbeing over time to major stressful events that entail trauma or grief. A growing body of evidence demonstrates the contribution of stress to mental health problems such as depression and anxiety (e.g., du Prel et al., 2024; Hussenoeder et al., 2024; Ross et al., 2017) as well as to physical health problems such as cardiovascular and gastrointestinal disease (e.g., Cairns et al., 2025; Ge et al., 2022; Leigh et al., 2023; Vaccarino and Bremner, 2024). Because of their prevalence and impact on health, understanding stress and how individuals regulate it is of great salience to health promotion, prevention of illness, the ability to cope with illness-related symptoms, and recovery.

However, challenges exist in the development of knowledge about stress that can inform improved health care. Among these is the fact that self-report of perceived stress has been a primary method for measuring stress. Clearly, an individual's experience of their own cognitive and emotional distress resulting from life stressors and their felt inability to cope with or control stressful circumstances provide invaluable information about stress. Numerous self-report measures are available that are cost effective and easy to administer; these factors foster their use. Nevertheless, the accuracy of self-report can be easily influenced. For instance, individuals may downplay stress or present themselves as more resilient than they feel because of perceived cultural or societal expectations. They may have difficulty accurately remembering and reporting stress experiences or may interpret items in a way that reflects their cultural or gender-based values. Some individuals have minimal introspective ability, resulting in a limited capacity to assess their feelings or thoughts accurately.

Biomarkers of stress represent an alternative and/or a complement to questionnaires that can help in addressing potential problems inherent in self-report. Biomarkers are objectively measured indicators of physiological, chemical, genetic and other biological processes that are implicated in the response to stress. They can be used to screen for or assess stress and enable us to identify and monitor potential changes in stress related to an exposure or intervention. Still, in considering their use, it is important to note that perceived stress and biomarkers of stress are not necessarily correlated (Epel et al., 2018; Leung et al., 2024; Sanogo et al., 2025). The absence of a predictable positive relationship between perceived stress and the biological response to stress may stem from a variety of factors. An individual's genetic predisposition can influence how sensitive the body is to particular stress biomarkers, resulting in exacerbated or diminished biological responses that diverge from the emotions or cognitive discomfort the person experiences (Buchanan and Lovallo, 2019; Soliva-Estruch et al., 2023). For instance, the body's stress pathways may be dysregulated by stressors even when the person feels emotionally stable or minimally distressed (Herber et al., 2025). Also, an individual's cognitive frame of mind regarding stress, learned coping skills, or quality of social support can moderate how they manage stress-related emotions. These factors may dampen or reduce the body's physiological response even when the stressor is identified as substantial (Epel et al., 2018; Palamarchuk and Vaillancourt, 2021). Further, biological responses to stress may change among individuals exposed to prolonged or chronic stress. They may experience habituation to stressors over time, resulting in diminished physiological or hormonal responses even when perceived stress is high (Barthel et al., 2025; Herman, 2013). Through all these mechanisms, the body's biological response to stress and the individual's perception of stress may be independent or discrete. In sum, not all biomarkers are triggered by perceived stress, nor does every experience that people perceive as “stressful” cause a biological response. There is growing evidence that the biological component of stress makes a unique contribution to understanding stress, yielding insights that are distinct from those associated with stress-related feelings and thoughts.

Because of its perceived salience to health and knowledge development, there has been a growing number of papers on stress biomarkers in the literature. Some have been systematic or scoping reviews focused on identification of studies that have used various biomarkers to assess stress and the knowledge found to date from this research (e.g., Noushad et al., 2021). Others have focused on an overview of common psychological and biological measures that are stress-related, providing brief summaries of each measure (e.g., Shah et al., 2024). Still others have covered stress biomarkers that derive data from one specific source, such as saliva or biosensors (e.g., Kim et al., 2023). Lastly, a few papers have reviewed markers associated with one particular biological system (immune system), age group (elderly), setting (occupational health), or stress-related concept (resilience) (e.g., Davis et al., 2023; Paribello et al., 2024). Although these various contributions are each important, our contrasting goal in this paper is to provide a “state of the art” review of key biomarkers being used to measure stress (Barry et al., 2022; Poddi and Rungatscher, 2026), with an emphasis on their conceptual and methodological underpinnings. Through this type of review, we seek to address pivotal points that shape the understanding and use of stress biomarkers from varied biological systems and sources. We describe the rationale for use of each marker as a measure of stress, discuss key methods involved in studying the marker, differentiate its use in measurement of acute vs. chronic stress, delineate how it can be used to assess resilience to stressors, highlight examples of the marker's use in research, and summarize its strengths and limitations. Our synopsis addresses biomarkers associated with the body's two primary stress response systems: the hypothalamic–pituitary–adrenal axis (cortisol, dehydroepiandrosterone) and the autonomic nervous system (heart rate variability, alpha-amylase) as well as immune system (cytokines and C-reactive protein), genetic (telomeres, DNA methylation), and multi-omic markers linked to stress (see Figure 1). We conclude with important considerations when using all stress biomarkers in research and recommendations for future research that can advance the field.

**Figure 1:**
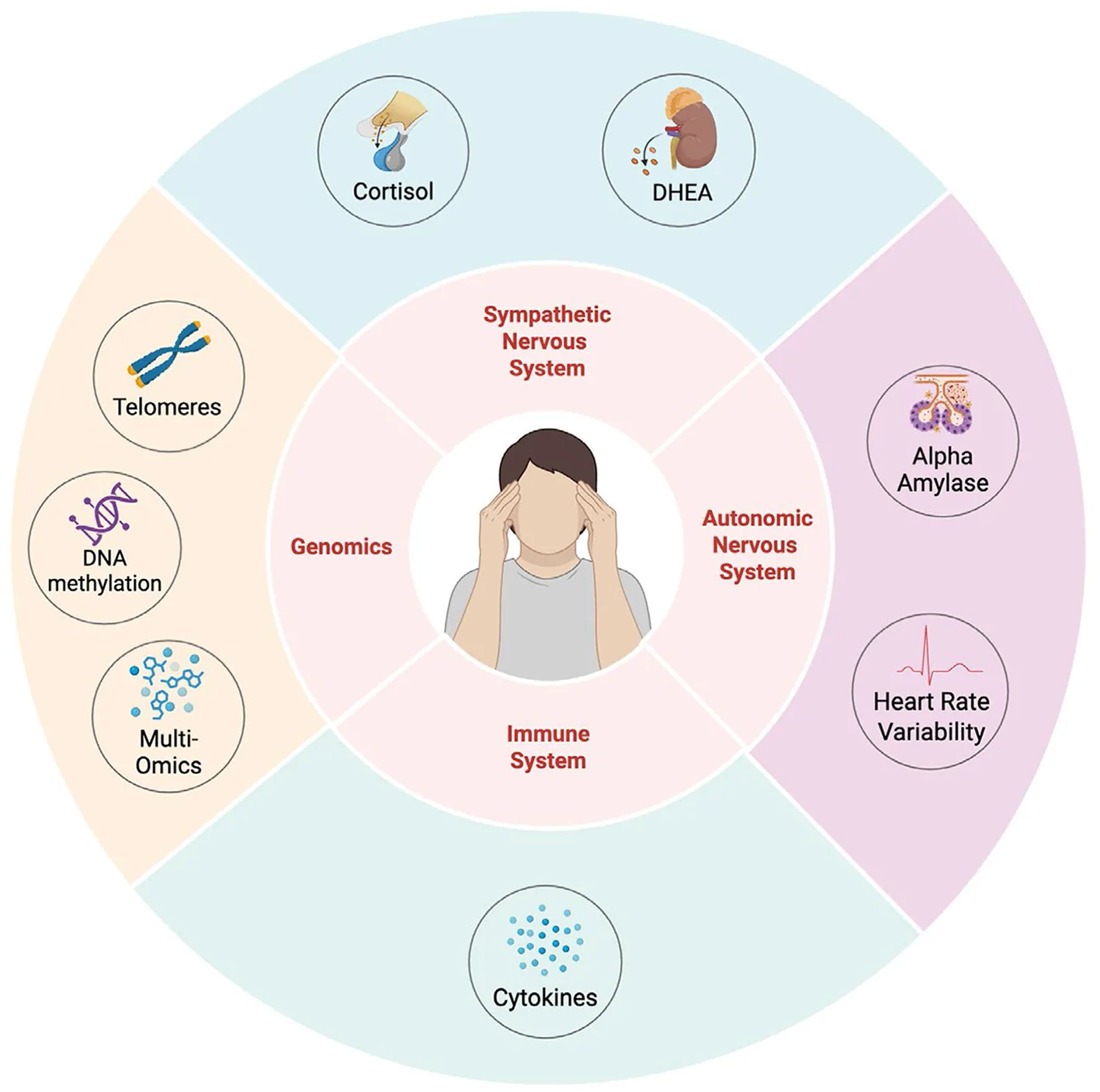
Key biomarkers associated with stress and their alignment with core biological systems.

The primary databases used to acquire relevant information for this review included Pubmed, Embase, and the Cochrane Library. We combined key word searches and citation tracking to identify relevant literature. Examples of terms used in the preliminary search were (“Stress” OR “Stressors”) AND (“Biomarkers” OR “Physiological Stress Response”). Terms used in specific biomarker searches were more targeted, such as (“stress” OR ”stressors”) AND (“alpha-amylase”). We focused on publications in the last 10 years but also incorporated seminal or foundational literature. Content specific to the review's goals (e.g., collection methods, research examples) was validated across publications to assure accuracy, timeliness, and breadth. Selection of appropriate content from this process relied initially on the expertise of the specific co-author responsible for addressing each biomarker. All authors then reviewed all content to assure its correctness, quality, comprehensiveness, and relevance.

## Markers specific to the hypothalamic–pituitary–adrenal axis

### Cortisol

Cortisol is a glucocorticoid hormone released as part of activation of the hypothalamic–pituitary–adrenal (HPA) axis in response to an acute threat or stressor (Engel and Gunnar, 2020; James et al., 2023; Kuhlman et al., 2017). It plays an essential role in stimulating diverse physiologic changes to cope with a stressor effectively, including gluconeogenesis, immune suppression, and increased metabolism (James et al., 2023). Cortisol release is regulated by a negative feedback system between the hippocampus and HPA axis to prevent excessive cortisol in the bloodstream and promote a return to allostasis once a stressor is removed (James et al., 2023; Khoury et al., 2019; Kuhlman et al., 2017). Excessive or repetitive HPA axis activation and failure to achieve allostasis, as in the case of chronic exposure to stressors, may stimulate persistent pathophysiologic processes that contribute to negative health outcomes (James et al., 2023; Khoury et al., 2019). This includes dysregulation of the stress response, which can manifest with either prolonged elevated or inadequate dampened cortisol secretion as a result of hyper- or hypo-reactivity of the HPA axis (Atkinson et al., 2016; Khoury et al., 2019; Kuhlman et al., 2017). Given that dysregulation of the HPA axis can affect various physiologic systems, cortisol concentration has been examined as a measure of stress in association with a number of physical and mental health symptoms and conditions (Braithwaite et al., 2016; Gilbert et al., 2017; James et al., 2023; Keeton et al., 2023, 2024; Khoury et al., 2019; Morris et al., 2017; Stalder et al., 2017; Weiss and Xu, 2024).

As a resting measure, cortisol can be used to determine an individual's baseline level prior to or following an acute stress response (Henckens et al., 2016), or it can be analyzed as an average level of stress over a period of time (Khoury et al., 2019). It also can be used to measure stress reactivity or recovery to assess the magnitude and duration of an acute response after exposure to a stressor (Atkinson et al., 2016; Dienes et al., 2019; Henckens et al., 2016; Puhakka and Peltola, 2020). HPA axis activity is largely affected by circadian rhythm and cortisol levels typically fluctuate throughout the day (James et al., 2023; Weiss and Xu, 2024). This variation in diurnal patterns affects the assessment of resting cortisol levels, often necessitating multiple and specifically timed measurements for accuracy (Atkinson et al., 2016; Weiss and Xu, 2024). The cortisol awakening response (CAR) is commonly used to evaluate the amount of change in cortisol from waking to approximately 30 to 45 min after waking, when it is known to be at its highest (Dienes et al., 2019). Diurnal cortisol slope, representing the change in cortisol from waking to evening bedtime, is also used as a measure of stress with flatter slopes associated with greater dysregulation and poor health outcomes (Adam et al., 2017). Cortisol reactivity and recovery can be assessed through multiple measurements before, during, and after exposure to a stressor, which usually involves structured experiments in a laboratory or similarly controlled environment (Dienes et al., 2019; Puhakka and Peltola, 2020). Another way to evaluate stress reactivity is using the area under the curve (AUC), which involves multiple cortisol measurements timed throughout the day to capture overall patterns of change in resting state (Atkinson et al., 2016). AUC is also used as an indicator of total hormone secretion across the day (Salomon and Weiss, 2024; Weiss and Xu, 2024). Research suggests that more resilient individuals have lower overall cortisol levels, a robust CAR involving a stronger spike when waking, a steeper cortisol slope or decline from waking to bedtime, and a more rapid return to baseline after experiencing a stressor (Aizpurua-Perez et al., 2023; Gao et al., 2025; Lai et al., 2020; Ringgold and Rohleder, 2025; Rösner et al., 2024).

Cortisol can be measured through a variety of biospecimens collected through minimally invasive techniques, making it a versatile biomarker for studies with populations across the lifespan. Acute stress can be measured through cortisol levels in saliva, urine, and blood (El-Farhan et al., 2017; Iqbal et al., 2023; James et al., 2023), and less commonly in skin (D'Agata et al., 2019) or sweat (Iqbal et al., 2023) specimens. Saliva is an ideal specimen due to its ease of collection (James et al., 2023), which facilitates the acquisition of multiple timed specimens and also makes it possible for participants to perform self-collection for greater convenience (Walls et al., 2020). Saliva also is thought to be a more accurate specimen for measurement of cortisol, because only the active fraction of the hormone passes through the parotid gland into the saliva (Iqbal et al., 2023). Chronic stress is often measured via hair specimens (e.g., Musana et al., 2020) as cortisol deposits into the hair and accumulates over time (Khoury et al., 2019; Stalder et al., 2017). Average hair growth is approximately one centimeter per month and hair cortisol concentration is demonstrated to be stable in samples up to three centimeters long, allowing for the measurement of average or chronic stress over a period of one to 3 months (Khoury et al., 2019; Stalder et al., 2017).

Liquid chromatography-mass spectrometry (LC/MS) is considered the gold standard for cortisol analysis due to its high sensitivity and specificity, although cost and the need for specialized analytic equipment can be barriers to its use (El-Farhan et al., 2017; Lee et al., 2024). Immunoassays offer a more accessible and affordable alternative, although they may be more vulnerable to cross-reactant interference that should be accounted for during analysis and interpretation (El-Farhan et al., 2017; Lee et al., 2024). Regardless of the analytic approach, certain factors are known to affect cortisol and should be considered during analysis and interpretation, including age, sex, and body mass index (James et al., 2023; Khoury et al., 2019). Cortisol is quantified in terms of nanomoles per liter (nmol/L) or micrograms per deciliter (mcg/dL), or in the case of hair specimens, picograms per milligram (pg/mg) (Gow et al., 2010; Khoury et al., 2019). One of the greatest limitations of cortisol as a stress measure is that although some have been proposed (de Kruijff et al., 2020; García-Blanco et al., 2016; Marceau et al., 2020; Noppe et al., 2014; Prado-Gasco et al., 2019), there are no agreed upon reference ranges specific to psychological stress (Iqbal et al., 2023). This is compounded by the fact that both elevated and blunted cortisol levels may be a sign of dysregulation, creating further challenges in interpretation (Atkinson et al., 2016; Khoury et al., 2019).

Cortisol demonstrates great versatility and utility as a measure of HPA axis function in diverse populations, including neonates (D'Agata et al., 2019; Weiss et al., 2023), adolescents (Leung et al., 2024; Prado-Gasco et al., 2019), pregnant people (Braithwaite et al., 2016; Keeton et al., 2024; Kim et al., 2020; Salomon and Weiss, 2024), and older adults, and across a range of racial and ethnic groups (DeSantis et al., 2015; Walls et al., 2020). In a study of 181 diverse infants and young (pre-school aged) children with low income, investigators examined associations between various socioeconomic stressor exposures and the children's cortisol levels (Tarullo et al., 2020). They found that, even after controlling for parental hair cortisol, higher hair cortisol concentration in the children was associated with lower parental education level and greater household food insecurity, indicating a chronic stress response to these persistent exposures. In this study they also analyzed children's acute stress response, using diurnal salivary cortisol measured at three timepoints over 3 days. Both age groups demonstrated a dysregulated acute stress response, albeit in different ways. In the infants, household chaos was associated with a higher AUC—reflecting elevated acute stress across the day—whereas in the young children, greater food insecurity was associated with a flatter diurnal slope—demonstrating persistent elevation and a delay in returning to baseline (Tarullo et al., 2020). This study is an excellent example of the different ways cortisol can be measured, analyzed, and interpreted to evaluate the impact of external stressors on the physiologic stress response.

### Dehydroepiandrosterone (DHEA/DHEA-S)

DHEA and DHEA-S (to which a sulfated molecule is attached) are the most abundant steroid hormones in humans (Neunzig and Bernhardt, 2014). They are produced by the adrenal glands and regulated by the HPA axis in response to the stress hormone adrenocorticotropic hormone (ACTH). In terms of their effects, DHEA and DHEA-S are highly correlated hormones. However, the sulfation process involved with DHEA-S makes it more stable and abundant in the body, with the majority of human circulating DHEA being the sulfated form (DHEA-S; Lin et al., 2025). It can be converted to DHEA when needed since DHEA more easily crosses the blood-brain barrier for immediate use. Even in the absence of stress, these hormones are present at measurable levels and serve as precursors to other hormones, including testosterone and estrogen. They are important neurosteroids that affect neurogenesis and neuronal survival as well as modifying the synthesis and release of neurotransmitters, among other functions (Starka et al., 2015). DHEA fluctuates throughout life, peaking around early adulthood and gradually declining with age; men appear to have higher levels than women (Chen et al., 2018; Samaras et al., 2013). Notably, DHEA serves as a glucocorticoid antagonist to stress hormones like cortisol, curbing their effects. As a result, DHEA and DHEA-S have been linked to resilience in response to stress (Girardi et al., 2024; Petros et al., 2013), stimulating more optimal emotions and mood that ultimately enhance longevity and good health (Jørgensen et al., 2021; Takeshita et al., 2025).

Because it fluctuates rapidly, DHEA is a better measure of acute stress response than is DHEA-S. Studies suggest that DHEA increases in response to acute stress, peaking at the end of a stressful event in its efforts to counteract effects of stress hormones like cortisol (Dutheil et al., 2021). In contrast, because of its longer half-life and stability over time, DHEA-S is a more optimal measure of overall baseline levels and hormonal status in response to chronic stress. DHEA-S decreases to lower baseline levels than the norm after prolonged stress (Lennartsson et al., 2022). The ability to produce DHEA-S appears to be decreased in individuals who experience higher persistent stress, including the potential for a blunted or diminished response to acute stressors over time (Lennartsson et al., 2015; Marcatto et al., 2024).

These hormones can be collected from various fluids, with research showing little difference in their effects or quality based on type of fluid used as the source (e.g., blood, saliva; Dutheil et al., 2021). Because it contains only the bioavailable, unbound fraction of hormones, saliva is typically best for DHEA (the free, unbound form of the hormone; Slettvoll et al., 2025). Samples should be refrigerated immediately and then frozen at −20 °C or colder if long term. Freeze-thaw cycles should be minimized since DHEA is sensitive to degradation. DHEA has a diurnal pattern, with higher levels in the morning and a decline across the day. It does not have the surge in hormonal level about 45 min after waking that occurs for cortisol but rather a gradual decline that starts at waking. As with other hormones having a circadian rhythm, the time of day when specimens are collected needs to be considered as a potential confound. Hair specimens can be used to assess chronic, sustained DHEA levels; time of day when collected is not a concern when using hair.

A variety of methods can be used to identify the overall concentration of DHEA or DHEA-S in a specimen, including Immunoassay, Radioimmunoassay (RIA), or Liquid Chromatography Coupled to Tandem Mass Spectrometry (LC-MS/MS). LC-MS/MS is considered the most accurate method (Boggs et al., 2016). However, Enzyme-Linked Immunosorbent Assay (ELISA) is commonly used because of its convenience and cost effectiveness. In addition to identifying the concentration or level of these individual hormones, researchers often examine the cortisol/DHEA-S ratio as a method of assessing HPA axis function. This ratio is calculated by dividing cortisol concentration by DHEA-S concentration in a specimen. A higher ratio is typically interpreted as less ability to regulate stress effectively, with cortisol having the potential for a more robust effect. By inverting the hormones in the formula (DHEA-S/cortisol), a higher ratio would suggest a stronger ability of DHEA-S to regulate cortisol and potentially adverse effects of stress.

Examples of research employing these biomarkers include studies by Takeshita et al. (2025) and Agorastos et al. (2023). It has been hypothesized that higher DHEA levels may slow the progression of biological aging. Takeshita and colleagues examined the degree to which DHEA-S from blood samples, cortisol, and the cortisol/DHEA-S ratio predicted epigenetic age and age acceleration of midlife individuals in the U.S., using a variety of epigenetic clocks. They found that the cortisol/DHEA-S ratio was a superior predictor of both epigenetic age and epigenetic age acceleration than either DHEA-S or cortisol alone. In the second study, Agorastos et al. built upon extensive research indicating HPA axis impairments in stress regulation as an underlying mechanism in depression. They assessed several HPA-axis related endocrine biomarkers (including DHEA and DHEA-S) in response to overnight HPA axis stimulation and suppression tests to better understand the potential role of these biomarkers in first-time vs. recurrent-episode depression of individuals with major depressive disorder (MDD). Based on salivary samples acquired over a 3-day period, researchers found that patients with recurrent episodes of depression had significantly lower levels of DHEA than patients experiencing their first depressive episode. Authors proposed that salivary DHEA may be a marker of individual stress resilience and progression of MDD.

A key strength is that DHEA and DHEA-S can be used to measure effective stress regulation and resilience in contrast to the many stress-related markers that focus on less optimal responses to stress. In addition, their validity as biomarkers has been shown when using varied types of biospecimens (blood, saliva, urine, hair) and they afford appropriate options for assessing acute stress response (DHEA) as well as basal or chronic stress response (DHEA-S). However, there can be significant variations in DHEA and DHEA-S levels between individuals, even after adjusting for known confounds. These variations can make their interpretation challenging as universal stress resilience indicators. In particular, sex and age differences in levels of the hormones need to be carefully considered when analyzing and interpreting data.

## Markers specific to the autonomic nervous system

### Alpha-amylase

Alpha-amylase (AA) is an enzyme produced in the salivary glands and the pancreas whose primary function is to break down starches and glycogen in the mouth as a first step in digesting carbohydrates. A secondary function is defense against bacteria in the mouth. However, it is also used as a key biomarker of stress. AA is a downstream enzyme associated with activation of the Sympathetic Nervous System (SNS), an interconnected, network of nerves and ganglia that enable the body's rapid response to stress. When the SNS is activated by stress, it stimulates the salivary glands to release norepinephrine which, in turn, increases AA secretion. Studies show that AA increases immediately in response to both physical and psychological stress (Ali and Nater, 2020), making it a valuable tool for assessing SNS reactivity in the acute response to stressors. But it is also used as a measure of basal SNS activation since it attenuates rapidly to environmentally-induced fluctuations, returning momentarily to baseline levels (Duchemin et al., 2015; Nater et al., 2013). There is also robust support for the stability of individual basal differences in AA (Out et al., 2013). Secretion patterns such as blunted AA levels at awakening and either hypersecretion or hyposecretion in response to stressors or across the day are typically viewed as indicators of ANS dysregulation (Ali and Nater, 2020; Kuras et al., 2017; Schneider et al., 2025). In contrast, lower diurnal α-amylase values and a moderate AA response to stressors are associated with greater resilience and an individual's more optimal stress management (Zapata et al., 2021). Research suggests that chronic activation of the SNS in response to stressors can lead to down-regulation of AA secretion, along with low basal AA activity and reduced AA reactivity in response to stressors (Mielock et al., 2017; Teixeira et al., 2015).

AA is assessed primarily in saliva (most common) and blood samples for purposes of research, although it is frequently sourced from urine for diagnostic purposes. Samples can be refrigerated for a short time (ideally a week at most) or aliquoted and frozen at −20 °C (up to 1 month) or −80 °C (up to 3 months). As with most biospecimens, repeated freeze-thaw cycles should be avoided to reduce risk of degradation. Commonly used methods to identify AA concentration include: (1) enzymatic assays that employ substrates which change color to quantify enzyme activity, and (2) spectrophotometry that measures the amount of light absorbed or transmitted by a solution to determine alpha amylase concentration (Erta et al., 2025). AA does have a diurnal pattern: levels decline within the first 30 min after awakening, then steadily rise during the day to peak in the late afternoon or evening. Studies suggest that a blunted decline on waking or a higher output during the day are indices of Autonomic Nervous System dysregulation (Ali and Nater, 2020; Jezova et al., 2020). Because of its diurnal pattern, it is important to control for time of specimen collection in research design, analysis, and/or interpretation of AA.

In addition to examining levels of AA across time (diurnal and longitudinal measures) or in response to a stressor, an AA/cortisol ratio has been examined as a marker of regulation or dysregulation in the symmetry of ANS and HPA axis function. Research indicates that higher values for this ratio are a better predictor of adverse stress experience and chronic stress than a cortisol/AA ratio, or individual AA and cortisol markers (Ali and Pruessner, 2012; Schneider et al., 2025).

Two illustrative studies that examined AA in the context of stress are discussed by Sawyers et al. (2024) and Zapata et al. (2021). Among other aims, Sawyers and colleagues examined genetic and environmental influences on AA stress reactivity to the Trier Social Stress Test (TSST). Their sample included twins as well as singletons from a general population of adolescents. Researchers measured salivary AA prior to the TSST and four times after the test, at intervals of 15 min. They found a substantial genetic influence on baseline and peak measures of AA but less influence on change from baseline to peak. Investigators suggest that these findings indicate most of the heritable influence on AA reactivity is determined by basal processes. Of interest, they also reported no significant relationship between measures of AA and cortisol, including no genetic overlap between these respective indicators of sympathetic nervous system and HPA axis arousal. In a very different type of study, Zapata et al. examined the association of emotional intelligence to AA (and cortisol) response of medical students during 4 different simulated mass casualty events that were part of resilience training for high stress situations. Salivary samples were acquired before and after each simulated scenario. Investigators found that lower AA predicted pre-event emotional intelligence, including more optimal stress management by students. Researchers proposed that higher AA may predict poor resilience potential in managing high stress situations and serve as a marker to identify students or other health personnel who may need specialized or additional training.

AA has notable strengths as a measure of stress. The enzyme enables assessment of sympathetic nervous system activity, which has received less attention in stress research than its companion stress response system—the HPA axis. AA can be used to assess either basal state or stress reactivity and can be non-invasively measured in saliva. In addition, the physiologic response of AA to a stressor is almost immediate, requiring no delay in specimen collection. However, this latter strength can also be a limitation since levels begin to decrease as soon as the stressor ends. As with many other biomarkers, AA can be influenced by factors such as age, lifestyle (e.g., diet, smoking), and environmental exposures so these must be considered in design and analysis. Similarly, methods of specimen collection (e.g., stimulating saliva by chewing vs. non-stimulated passive drooling), storage conditions, and type of assay can influence results (Ali and Nater, 2020).

### Heart rate variability

Heart rate variability (HRV) reflects fluctuations in the time intervals between successive heartbeats. It is regulated by the autonomic nervous system (ANS) and comprises the sympathetic nervous system (SNS), which accelerates heart rate during stress, and the parasympathetic nervous system (PNS), which slows it for recovery. The dynamic interplay between these systems helps maintain physiological homeostasis. HRV serves as a non-invasive measure of autonomic regulation in response to stress, including impairments or resilience in SNS or PNS functioning (Shaffer and Ginsberg, 2017).

In less nuanced studies, HRV measurement often relies solely on measurement of PNS activity, often referred to as “vagal tone.” Vagal tone is viewed as the degree of activity exerted by the vagus nerve on the heart, which slows the heart down via acetylcholine release at the sinoatrial node (Laborde et al., 2017). When considering this singular measure of HRV, higher HRV generally signifies strong parasympathetic activation and effective stress recovery, while low HRV suggests reduced parasympathetic activity and less effective regulation of stress. Based on this global measure of HRV, acute stress typically results in a temporary reduction in HRV due to heightened sympathetic activation and decreased parasympathetic activity, preparing the body to cope with immediate challenges (Jiryis et al., 2022; Kim et al., 2018). These changes result in heightened cardiovascular reactivity and metabolic demand, creating a state of physiological arousal. Conversely, chronic stress is associated with persistently low, parasympathetic HRV, indicating impaired autonomic flexibility and long-term dysregulation of the stress response (da Estrela et al., 2021). Longitudinal studies have shown that individuals with chronically low parasympathetic HRV are more susceptible to health problems (Chalmers et al., 2014; Kubota et al., 2017), underscoring the importance of HRV as a predictive marker of stress-related disease risk. In contrast, increased HRV-related parasympathetic activity is associated with improved autonomic regulation, facilitating physiological restoration. In sum, when using this parasympathetic measure of HRV, higher HRV suggests greater autonomic flexibility and resilience (An et al., 2020; Perna et al., 2020), while lower HRV indicates impaired stress adaptability and is associated with increased disease risk (Heiss et al., 2021).

However, in more precise, detailed studies using HRV, many sophisticated metrics are used to examine HRV. These are primarily assessed through electrocardiography (ECG), which records the heart's electrical activity and provides explicit measurements of the time intervals between successive heartbeats (R–R intervals). In research seeking finer HRV distinctions, it is commonly assessed using time-domain or frequency-domain measures (Shaffer and Ginsberg, 2017). Time-domain measures evaluate the time period between successive heartbeats. Key metrics include the standard deviation of NN intervals (SDNN), which reflects overall HRV influenced by both SNS and PNS activity, and the root mean square of successive differences (RMSSD), which is primarily associated with parasympathetic modulation and vagal tone. Another commonly used time-domain measure is the percentage of NN intervals differing by more than 50 milliseconds (pNN50), serving as an index of vagal activity. In contrast, frequency-domain analysis involves spectral decomposition of HRV signals to differentiate SNS and PNS activity and is typically performed using Fast Fourier Transform (FFT) or wavelet analysis. This approach categorizes HRV components into distinct frequency bands, including low-frequency (LF) power, high-frequency (HF) power, and the LF/HF ratio. LF power is typically interpreted as reflecting a complex mix of both PNS and SNS or baroreflex activity, while HF power is predominantly associated with parasympathetic influence. The LF/HF ratio is often used as an indicator of autonomic balance, with higher values suggesting increased sympathetic dominance and lower values indicating parasympathetic dominance. However, caution is needed when interpreting this ratio since there are mixed findings regarding its physiologic meaning (Gullett et al., 2023).

While electrocardiography (ECG) has been the traditional gold standard for measuring HRV, newer technologies such as photoplethysmography (PPG) and chest strap monitors have emerged (Mirescu and Harden, 2012; Plews et al., 2017). The various technologies have different advantages and limitations. ECG measures the electrical activity of the heart through electrodes placed on the skin, offering high accuracy in detecting HRV metrics. However, ECG systems are generally used in clinical or controlled environments due to their complexity and the need for professional interpretation. Photoplethysmography (PPG), commonly found in wearable devices, detects blood volume changes in the skin using light. While PPG offers a convenient and user-friendly alternative to ECG, it is generally less precise for HRV measurement, especially for frequency-domain analyses (Charlton et al., 2023). Chest strap monitors, often equipped with ECG sensors, provide real-time heart rate data with high accuracy and are widely used in sports and fitness applications but may be less comfortable to wear.

While HRV is widely assessed using photoplethysmography (PPG) and chest strap monitors in older children and adults, these methods have limitations for infants. Wearable devices such as Oura Ring 4 is sized for individuals with ring sizes between 4 and 15 while the Fitbit Ace LTE smartwatch is designed for children older than 7 years old. The Polar H10 chest strap has been validated for HRV measurement in young children (ages 3–5) (Speer et al., 2020), but few studies have examined HRV in infants using reliable methods. Recent studies demonstrate that HRV, assessed with electrodes on the skin, is a feasible measurement even in infants when using electrocardiography (ECG). We successfully recorded HRV in infants at 1, 6, and 12 months of age in response to a standardized stressor (Weiss et al., 2024a,b). Using frequency domain measurements of HRV, we examined the effects of prenatal stressors on autonomic nervous system development as well as the role of HRV in infant stress regulation. Our studies highlight ECG as a viable and effective method for assessing HRV in infancy, enabling research on early-life stress and autonomic regulation as early as the first month of life.

HRV offers several advantages as a stress biomarker. It is non-invasive and cost-effective, as HRV can be assessed using ECG or wearable devices, allowing for real-time monitoring. HRV is sensitive to autonomic changes, capturing dynamic shifts in ANS activity and making it a valuable tool for stress assessment. It is applicable across various contexts, including various psychological, occupational, and clinical settings. However, HRV has limitations. It is influenced by multiple factors such as age, physical fitness, circadian rhythms, and medication use, necessitating careful interpretation. HRV measurement in infants and children presents unique challenges, especially in controlling artifact related to movement. Lastly, variability in measurement protocols, including differences in recording duration and posture, can impact HRV outcomes and limit cross-study comparability.

## Immune system markers

### Cytokines and C-reactive protein

Inflammatory biomarkers have become increasingly valuable for understanding the biological underpinnings of stress. Stress, whether acute or chronic, activates neuroendocrine systems that influence immune function. One critical pathway involves the activation of the hypothalamic–pituitary–adrenal (HPA) axis and the sympathetic-adrenal-medullary (SAM) system, leading to the release of stress hormones such as cortisol and catecholamines (Chu et al., 2025). These hormones interact with immune cells to modulate inflammatory processes, triggering the release of pro-inflammatory cytokines such as tumor necrosis factor-alpha (TNF-α), interleukin-6 (IL-6), and interleukin-1 beta (IL-1β), as well as acute-phase reactants like C-reactive protein (CRP) which is produced in response to cytokines (CRP; Chu et al., 2025; Slavich and Irwin, 2014). Cytokines and CRP serve as quantifiable indicators of the body's physiological stress response.

The immune system's response to stress operates on a continuum from adaptive to maladaptive, depending on the nature, duration, and intensity of the stressor. An adaptive, resilient immune response has usually been associated with less inflammatory activity, such as lower levels of TNFα and IL-6 (Ringgold and Rohleder, 2025; Ryan and Ryznar, 2022). An anti- inflammatory profile has been proposed as a mechanism contributing to psychological resilience (Paribello et al., 2024). Acute stress, such as a time-limited public speaking task or competitive examination, often results in a transient and reversible increase in inflammatory cytokines (Yamakawa et al., 2009). This response is thought to prepare the body for potential injury or infection and typically resolves once the stressor is removed (Marsland et al., 2017). In contrast, exposure to chronic stressors, such as caregiving, interpersonal violence, or sustained poverty, can lead to persistent dysregulation of inflammatory pathways. One mechanism underlying the effects of chronic stress is glucocorticoid resistance, whereby immune cells become less responsive to cortisol's anti-inflammatory effects. Glucocorticoid resistance results in heightened cytokine production even in the presence of elevated cortisol levels (Marwaha et al., 2025). Over time, this low-grade, systemic inflammation can have widespread effects on physical and mental health, contributing to the development and progression of depression, anxiety, cardiovascular disease, autoimmune disorders, and metabolic dysfunction (Furman et al., 2019). Thus, inflammatory biomarkers offer a mechanism by which psychosocial stress becomes biologically embedded and translates into long-term health outcomes.

A variety of biospecimen collection methods have been developed to measure inflammatory biomarkers, each suited to different research contexts. Venipuncture is the most commonly used and yields plasma or serum, which allows for high-sensitivity quantification of cytokines, including TNF-α, IL-6, IL-1β, and CRP. However, blood draws are invasive, require trained personnel, and may induce additional stress in participants, especially among children or individuals with a phobia to needles. To overcome these challenges, researchers have increasingly adopted alternative, less invasive collection methods. Dried blood spots (DBS), obtained via finger prick, provide a viable alternative that has been validated for use with some inflammatory markers, including TNF-α and CRP (Reed et al., 2024; Samuelsson et al., 2015). DBS samples can be collected in participants' homes, stored at room temperature, and mailed to laboratories without needing to be frozen immediately, making them ideal for large-scale, field-based, or longitudinal studies (Ostler et al., 2014). Saliva sampling is another non-invasive method, particularly in pediatric (Fey et al., 2024; Leung and Weiss, 2025) and psychiatric research (Szabo and Slavish, 2021). Salivary cytokines such as IL-1β, IL-6, and TNF-α can reflect both localized and systemic inflammatory activity (Rathnayake et al., 2013; Szabo and Slavish, 2021). While salivary measures are easier to collect and impose less participant burden, they may be more susceptible to variability due to oral health, time of day, and food intake (Riis et al., 2021). Emerging methods, such as inflammatory marker detection in hair and urine, offer promising alternatives for assessing long-term inflammatory exposure, though these techniques are less established and require further validation. It is important to note that CRP and cytokines differ in their stability. CRP is very stable. It can be stored at room temperature or refrigerated (4 °C) without significant degradation. In contrast, cytokines are often present in low (pg/mL) concentrations and require more immediate processing; they also need to be kept chilled or frozen at −80 °C during long-term storage to prevent degradation.

Empirical studies have demonstrated the utility of inflammatory biomarkers in capturing both acute and chronic stress responses across diverse populations. For example, the Trier Social Stress Test (TSST), a standardized laboratory stressor involving public speaking and mental arithmetic, has been shown to elicit rapid increases in IL-6 and TNF-α within 30 to 90 min post-stressor (Steptoe et al., 2007). In contrast, chronic stress has also been linked to sustained elevations in inflammatory markers. Kiecolt-Glaser et al. (2003) found that older adults caring for a spouse with dementia, a chronic stressor, showed a significantly greater increase in plasma IL-6 levels over 6 years compared to non-caregivers. Elevated IL-6 levels persisted even after caregiving ended, supporting IL-6 as a biomarker of chronic stress with long-term health implications. Similarly, in a longitudinal study of adolescents, participants who faced recent stressful events and showed greater increases in inflammation (IL-6 and CRP) were more likely to develop severe depressive symptoms after 1 year, suggesting that the combination of stress and heightened inflammatory responses predicts increased depression risk (Kautz et al., 2020). These research findings underscore the sensitivity of inflammatory biomarkers to both environmental exposures and psychological experiences, reinforcing their value as tools for stress assessment.

While inflammatory biomarkers offer several advantages. They can provide mechanistic insight into how stress affects physical health by mapping the pathways between psychological experiences and immune function. Additionally, technological advancements have made biomarker assays more affordable, scalable, and sensitive, allowing for their integration into large epidemiological studies and clinical trials. However, several limitations must be considered. Inflammatory biomarkers are not specific to stress; they can be influenced by numerous confounding factors, including age, sex, socioeconomic status, body mass index, substance use, medications, and medical conditions. This lack of specificity necessitates careful control of covariates and standardization of sampling protocols (Ain et al., 2021). Another limitation is the temporal variability of cytokine levels; because they fluctuate throughout the day and in response to acute events, timing of collection must be tightly controlled (Nakao, 2014).

## Genetic markers

### Telomeres

Telomeres are nucleoprotein structures that serve as protective caps at the ends of chromosomes and play a critical role in maintaining genomic stability (Srinivas et al., 2020). The telomere complex is comprised of connected, repeated DNA sequences bound by a set of proteins (Blackburn et al., 2015). As cells naturally divide, the protective telomeres shorten, limiting the number of times a cell can replicate and, ultimately, resulting in cell death or senescence. However, an enzyme called telomerase adds DNA sequences to the ends of chromosomes to counteract the shortening of telomeres, helping to maintain their length and prolong the life of the cell.

In addition to serving as a general marker of cellular integrity and aging, telomere length is a specific indicator of exposure to stress. Both stressful events and perceived stress have been associated with telomere shortening (Rentscher et al., 2020). Acute psychological stressors can induce dynamic, short-term changes in telomerase activity, although these effects appear transient rather than indicative of long-term telomere shortening (Epel et al., 2018; Müller-Alcazar et al., 2024). Cumulative effects of chronic stress are more enduring (Libertini et al., 2021). Under conditions of persistent stress, telomerase suppression and telomere attrition are driven by chronic activation of the hypothalamic–pituitary–adrenal (HPA) axis and its related effects on cortisol, oxidative stress, and inflammation. Conversely, psychological and psychosocial resilience have been associated with longer telomere length and greater telomerase activity (Johnson et al., 2019; Puterman and Epel, 2012).

Beyond the effects of stress on telomere erosion within the individual experiencing stress, a growing body of evidence indicates that prenatal maternal stress exposure may be transferred intergenerationally, influencing child telomere length and related adverse health outcomes across the lifespan (Entringer et al., 2018; Moshfeghinia et al., 2023; Van den Bergh et al., 2020). In addition, maternal resilience during pregnancy has been positively associated with newborn TL (Verner et al., 2021).

Telomere length is most frequently measured by extracting DNA from venous whole blood to assess peripheral blood leukocytes (white blood cells). However, minimally invasive methods like saliva, buccal swabs, or dried blood spots from finger pricks are increasingly used to collect telomere samples and have shown good validity and reliability (Rej et al., 2021). There are several common techniques for telomere measurement: Southern blot analysis (Terminal Restriction Fragment; TRF), qPCR (Telomere/Single-copy gene ratio; T/S Ratio), fluorescence *in situ* hybridization (FISH) methods (length derived from imaging), and single telomere length analysis (STELA) (Yu et al., 2024). Emerging long-read, sequencing approaches are gaining increasing popularity (Coulter et al., 2024). These methods vary in precision, scalability, and feasibility for large-scale studies and are highly dependent on the research question at hand.

Using these techniques, three different metrics can be determined for telomere length: absolute (base pairs), relative (ratio), or average (mean) length. Absolute telomere length provides the exact number of base pairs and is measured using techniques like TRF, STELA, and long-read sequencing. TRF, the gold standard, offers high accuracy but requires large DNA samples, is labor-intensive, and unsuitable for large studies. STELA detects short telomeres with high resolution but is capable of processing only a small amount of data within a given timeframe and is limited to specific chromosomes. Long-read sequencing enables absolute telomere length measurement across individual chromosome ends, with methods like Telo-Seq and Telometer capturing genome-wide telomere heterogeneity with a large amount of data in short periods (Coulter et al., 2024; Schmidt et al., 2024). Relative telomere length is commonly measured using qPCR and provides a T/S ratio by comparing the telomere signal (T) to a reference gene signal (S) (Lin et al., 2019). While it does not provide exact base pair length, qPCR is cost-effective and scalable, making it ideal for large epidemiological studies. Average telomere length reflects the mean telomere length across chromosomes or cells. It can be assessed using TRF, qPCR, or FISH. FISH uses fluorescent probes to measure telomeres at the level of individual chromosomes and cells, aiding research into specific disease processes (Yu et al., 2024).

Telomeres are primarily used to assess varied types of chronic stress (e.g., psychological, social, physical and environmental) and indicate the extent of cellular aging associated with specific stressors. For example, Incollingo Rodriguez et al. (2022) examined the relationship between psychosocial stressors, telomere length, and the risk for postpartum depression. One hundred fifty Latine/x mothers were followed during pregnancy to assess stressors and mental health outcomes across the postpartum period. Findings suggest that acculturative stress during pregnancy was associated with shortened telomere length (assessed via qPCR from venous blood samples), which was also associated with higher risk of postpartum depression. Another example is a study to determine whether material hardship (e.g., family's inability to pay for health care, rent, or food; homelessness) was associated with shorter salivary telomere length among children in a longitudinal birth cohort at ages 9 and 15 (Moeckel et al., 2024). Absolute telomere length was assessed from salivary DNA of 1990 children using qPCR. Material hardship experienced during infancy and toddlerhood was associated with shorter telomere length at both later ages, indicating the impact of early economic stressors on cellular mechanisms associated with stress.

Telomere length is a valuable biomarker for stress research. It can be measured using accessible, minimally invasive methods, but is sensitive to external factors such as age, lifestyle, and environmental exposures (Ahlers et al., 2024). Due to its sensitivity to modifiable factors, research has shown that stress management and health behavior interventions (e.g., mindfulness, physical activity, diet, sleep) can reduce stress-related telomere shortening and related disease risk (Barragán et al., 2021; Deng et al., 2016; Epel and Prather, 2018; Qiao et al., 2020; Schutte et al., 2020; Shalev et al., 2013). Various approaches to telomere measurement have strengths and limitations. Absolute telomere length offers the most precision, while relative and average measurements can be more practical for large-scale studies, balancing accuracy, cost, and feasibility. The choice of method depends on research focus, sample availability, and need for precision or scalability. In summary, telomere length serves as a crucial biomarker for understanding the biological effects of chronic stress, offering valuable insights into cellular aging, disease susceptibility, and the potential benefits of stress-reducing interventions.

### DNA methylation

DNA methylation is a reversible covalent addition of a chemical tag called methyl groups (CH_3_) to the DNA base molecule with or without cell division (Angeloni and Bogdanovic, 2019). DNA methylation has emerged as a key biomarker for stress due to its ability to regulate gene expression in response to stressors. Stressors can produce alterations in DNA methylation patterns, particularly in the hypothalamic–pituitary–adrenal (HPA) axis regulatory genes (Humphreys et al., 2019). The significance of DNA methylation as a marker for stress has gained attention, in part, because it predicts both short- and long-term outcomes of stress. For instance, a study employing the Trier Social Stress Test revealed a significant correlation between a 10-min stress exposure and hypermethylation of the oxytocin receptor and brain-derived neurotrophic factor; the correlation diminished after 90 min of acute stress exposure (Unternaehrer et al., 2012). Long term stress effects of DNA methylation have been shown in varied longitudinal research (Bakusic et al., 2017; Chang et al., 2024; Comes et al., 2020; Harms et al., 2017; Rutten et al., 2018; Mulder et al., 2021; Urdinguio et al., 2016). For example, a longitudinal analysis of deployed military servicemen revealed that time-dependent decreases in DNA methylation at specific genomic regions (ZFP57, RNF39, and HIST1H2APS2) were significantly associated with increasing PTSD symptoms.

As methodologies for epigenetic analysis continue to advance, DNA methylation profiling promises improved precision in stress research, facilitating early risk detection and informing personalized interventions (Li and Tollefsbol, 2021; Sherpa et al., 2024). DNA methylation can be assessed using samples from saliva, blood, buccal swabs, hair follicles, or placenta. The chosen sampling source depends on the study design, research objectives, funding, feasibility, and participants' perceptions and responsiveness to different methods (Singer, 2019). Saliva and buccal swabs are commonly used as noninvasive, self-collected DNA methods that yield high-quality DNA comparable to blood samples for genomic applications (Garbieri et al., 2017; Hu et al., 2012). DNA is extracted in the laboratory using denaturing chemicals such as phenol-chloroform (Gupta, 2019) or by more recent techniques such as silica matrices and magnetic bead-based extraction (Jansson et al., 2024). The gold standard for methylation analysis is bisulfite conversion that differentiates methylated from non-methylated bases (Jeddi et al., 2024). There are several platforms for methylation analysis, such as PCR, sequencing, or array-based approaches, depending upon whether the focus is site-specific, regional, or whole genome analysis (Bendixen et al., 2023; Kumar et al., 2020; Ramalho-Carvalho et al., 2018; Wu and Kuan, 2018). Methylation-specific PCR is a targeted approach to identify DNA methylation at specific CpG sites subsequent to bisulfite conversion (Bendixen et al., 2023). Other high-throughput platforms used in wider methylation analysis are BeadChip arrays and Whole-Genome Bisulfite Sequencing (Agius et al., 2023; Gu et al., 2021; Wu and Kuan, 2018). Data normalization and quality control are essential to minimize bias and ensure that the methylation data mirrors true biological variances (Singer, 2019).

Scientists have deployed DNA methylation in stress-related research to uncover the biological mechanisms underlying stress response mechanisms and their long-term health implications. Targeted studies focusing on stress-responsive genes, such as NR3C1 (glucocorticoid receptor), FKBP5 (a key regulator of the stress response; Grasso et al., 2020), and SLC6A4 (serotonin transporter; Jiang et al., 2019), have revealed specific DNA methylation changes linked to stress exposure. For example, Wilker et al. (2023) demonstrate the use of targeted DNA methylation analysis and found that conflict survivors exhibited a significant increase in methylation at cg25535999 within NR3C1 post 4 and 10 months of narrative exposure therapy compared to their baseline (pre-treatment) level. Epigenome-wide association studies (EWAS) are also powerful tools for identifying DNA methylation changes associated with environmental exposures, including psychosocial stress (Viggiano and de Pinto, 2017). The study by Sharma et al. (2022) exemplifies an EWAS approach applied to prenatal stress, identifying five differentially methylated CpG sites in newborn saliva significantly associated with prenatal psychosocial stress.

Overall, DNA methylation is useful for assessing the epigenetic impacts of stress because it is an accurate measure of dynamic gene regulation in response to environmental experiences (Jiang et al., 2019; Zhang and Liu, 2022). Research shows that DNA methylation is an early indicator of stress-related pathologies preceding clinical manifestations, which makes DNA methylation a favorable tool for disease detection and early intervention (Lussier et al., 2024). In addition, DNA methylation can be used to determine signatures of psychological resilience in response to stress (Lu et al., 2023; Smeeth et al., 2021). However, the presence of methylation (or its absence) cannot be considered as an indicator of resilience overall. Whether it is a measure of resilience will depend on the specific genes which are methylated, the effects on gene expression, and the function of these genes on health and wellbeing in the face of stress. Methylation patterns also provide useful insight into intergenerational stress outcomes and suggest applications in personalized medicine. Finally, a prominent advantage of DNA methylation as a stress marker is its potential for reversibility (Zhang and Liu, 2022). Epigenetic modifications can be reversed by therapeutic modalities, providing an important marker of stress-related change for intervention studies, with implications for treatment and alleviation of stress-related ailments.

However, DNA methylation is less ideal for measuring immediate responses to acute stress due to its relatively slow changes (Hing et al., 2014). Methylation changes are tissue and cell specific, and findings from blood or saliva may not fully reflect changes in the brain; hence, the results need to be considered in a tissue or cell context (Zhang and Liu, 2022). Additionally, DNA methylation is influenced by confounding factors such as age, sex, race, smoking, and diet, which complicate the isolation of methylation's direct stress effects from other factors (He et al., 2017; Zhang and Liu, 2022). Further, laboratory methodology to measure methylation is challenging; assays are complex, expensive, and require experienced personnel for credible outcomes (Li and Tollefsbol, 2021; Sherpa et al., 2024). Finally, the effect size of stress-related DNA methylation is often small, necessitating larger sample sizes for meaningful conclusions (Welsh et al., 2023). Still, while not a stand-alone diagnostic tool, DNA methylation holds significant potential for precision health, helping in the potential development of targeted interventions for individuals at risk for stress-related disorders.

## Multi-omics

Multi-omics is the integration of molecular biomarkers from two or more -omics layers (e.g., genomics, epigenomics, transcriptomics, proteomics, metabolomics, microbiomics) (Hasin et al., 2017). A complement to traditional biomarkers that commonly measure stress-related pathology respective to a single physiological system, multi-omics offer systems-level insights into upstream dysregulation influencing downstream outcomes, revealing stress-responsive networks and latent perturbations that may precede overt pathology (Karczewski and Snyder, 2018). While the flexibility of this approach is a notable strength, it also introduces significant methodological variability. As such, a comprehensive review of the full range of -omics layers and methods is beyond the scope of this paper. Instead, we provide a high-level overview of each -omics layer.

Each -omics domain represents a distinct layer of biological information, forming a hierarchical framework that flows from upstream genetic information to biomarkers that reflect functional activity (Hasin et al., 2017). At the foundation, genomics captures the DNA sequence (genes) inherited at conception, which provides a relatively stable blueprint for how cells can function within the body across the life course. Modifications to this blueprint (e.g., DNA methylation, telomere length) are captured in epigenomics, as discussed in preceding sections. These two layers determine what biological instructions are transcribed into the RNA that will be translated into proteins, broadly referred to as gene expression (DNA ➔ RNA ➔ protein). The transcriptome measures the messenger RNAs (mRNAs) produced during the transcription stage of gene expression, as well as small non-coding RNAs (miRs) that regulate gene expression post-transcriptionally, modulating mRNA translation. The functional products of gene expression (proteins) are captured in proteomics, providing insight into the molecules carrying out many of the biological processes underlying human health and disease. The small molecules (metabolites) generated from biological processes (e.g., protein functions, stress responses) are captured in metabolomics, revealing a snapshot of biological activity and physiological states. Distinct from these layers measuring host (human) biology, microbiomics characterizes the microbial ecosystem residing in or on the human body that interacts with host physiology. When integrated, these layers provide multisystem insights into the coordinated processes and biological interactions shaping human physiology under normal conditions and in response to stress.

Biomarker measurement is determined by research context and objectives, as -omics layers differ in their responsiveness to stress (e.g., acute vs. chronic) and biospecimen sources differ in their ability to measure systemic (e.g., blood) vs. localized (e.g., fecal-gut) stress responses (Longoria et al., 2025; Mengelkoch et al., 2024). To illustrate study design considerations for biomarker selection, we provide a practical example focused on integrating layers relevant to the gut-brain-axis (GBA), a stress-responsive network linking the gut and the brain through bidirectional communication across multiple pathways (e.g., neural, neuroendocrine, immune, metabolic) (Cryan et al., 2019; Cryan and Dinan, 2012). Our example will focus on inter-dynamics among the microbiome, the metabolome and the transcriptome. Profiling of the gut microbiome is one of the most common layers used to study gut-related responses to stress, which is typically achieved through sequencing (analyzing) microbial DNA extracted from fecal specimens using methods such as 16S rRNA gene sequencing or shotgun metagenomics (Cryan et al., 2019). While identifying which microbes are present provides important information about gut microbial composition and relationships with stress, it offers limited information about the functional activity of these microbes and interactions with host physiology (i.e., GBA signaling). Fecal-derived metabolites provide insight into coordinated responses (microbial-host) to stress within the gut prior to systemic absorption and blood-derived metabolites provide insight into metabolites that have entered systemic circulation (Nicholson et al., 2012; Visconti et al., 2019). Therefore, metabolomics is a complementary layer that can reveal signaling molecules influencing host physiology in response to stress. Given GBA signaling involves interconnected pathways (e.g., neural, neuroendocrine, immune, metabolic), the profiling of circulating microRNA transcriptomes (plasma-derived miRs) can serve as a valuable approach to identify upstream regulatory responses to stress (e.g., gene targets, enriched pathways) (Issler and Chen, 2017). Notably, although circulating miRs are not inherently tissue specific, computational methods (e.g., tissue-enrichment databases) or different sequencing approaches (e.g., extracellular vesicle-derived miR profiling) can be applied to approximate signals from specific tissue origins (e.g., gut, brain) (Keller et al., 2022; O'Brien et al., 2020; Wiegand et al., 2017). This example of an integrative framework is merely one of many that could be applied for biomarker discovery and advancing multisystem perspectives of stress physiology.

Evidence from multi-omics research has been used to quantify an individual's biological resilience in response to stressors (Yamada, 2026). This approach integrates multi-omics data to model the dynamic equilibrium between biological damage and the capacity to repair and regain function (resilience). It captures a system's capacity to recover, reorganize, and maintain homeostasis under stress (Ruden, 2025).

However, the major focus in the field is on the adverse effects of stress. Multi-omics approaches are increasingly being used in research to identify new biomarkers of stress and stress-related outcomes [e.g., mood disorders, post-traumatic stress disorder (PTSD)], with goals to improve diagnosis and treatment across diverse populations as well as advance multidimensional frameworks of disease development (Hasin et al., 2017; Karczewski and Snyder, 2018). For instance, as part of a Department of Defense initiated multi-site Post-Traumatic Stress Disorder (PTSD) Systems Biology Consortium, Dean et al. (2020) applied multi-omics methods to validate a multi-omics panel of blood-based biomarkers to diagnosis combat-related PTSD. By including biomarkers from multiple -omics layers (e.g., epigenomics, transcriptomics, metabolomics), this panel not only performed well in detecting PTSD cases from combat-exposed controls (81% accuracy) but also revealed multisystem dysregulation signaling common PTSD-related comorbidities. This study highlights the transdiagnostic potential and clinical efficiency of multi-omics biomarkers of stress. When applied to research examining prenatal stress, multi-omics methods have also played an integral role in advancing the Developmental Origins of Health and Disease (DOHaD) framework (Lapehn and Paquette, 2022; Mengelkoch et al., 2024). Integrative analyses of placental biomarkers (e.g., epigenomic, transcriptomic, metabolomic) have revealed molecular signatures linking prenatal stress exposures to placental alterations in neuroendocrine, immune, and metabolic pathways involved in fetal development, as well as neurobehavioral outcomes in offspring (Freedman et al., 2022; Santos et al., 2020). Together, these applications highlight the value of multi-omics approaches in elucidating how stress exposures contribute to multisystem dysregulation and shape health trajectories across the life course, as well as their translational potential for improving risk stratification, diagnosis, and tailored intervention of stress-related health outcomes. Nonetheless, realizing this potential will require overcoming key challenges related to sequencing costs, the computational intensity required to process and integrate multiple large-scale datasets, and the large sample sizes needed to achieve adequate power in integrative analyses (Mengelkoch et al., 2024). Although rapid advances in -omics technologies are steadily reducing costs of data generation, expenses associated with biomarker quantification, data storage, and analytic infrastructure remain substantial (Misra et al., 2019). Overcoming these limitations remains essential for translating multi-omics findings into clinically actionable biomarkers and scalable tools for routine care in clinical practice today.

## Conclusions and recommendations

### Considerations when studying biomarkers of stress

Each of the biomarkers discussed in this paper has significant potential to expand knowledge of stress. Selection of the appropriate biomarker to use should be based primarily on the specific research aim and the underlying mechanistic pathway of interest. However, the timespan of stress being assessed must also be considered. As indicated in discussion of the various biomarkers, some are better measures of acute stress than others based upon how rapidly they are activated and can capture immediate or transient responses. In contrast, other markers gauge longer-term, sustained or cumulative stress (often involving downstream changes) and may be better measures of chronic stress. The suitability of each biomarker for measuring acute vs. chronic stress is detailed in Table 1. Figure 2 highlights the *primary* use of the different biomarkers for measurement of acute and chronic stress. As shown by the color differences in the figure, some markers are primarily used to assess acute stress (gold) while others more often serve as measures of chronic stress (blue). Still other biomarkers frequently serve as indicators of both (green).

**Table 1:** Key advantages, limitations, and timeframe considerations in using various stress biomarkers.

System	Biomarker	Key advantages	Key limitations	Suitability for acute and chronic stress
HPA axis	Cortisol	- Versatile for use across lifespan. - Allows for measurement of acute or chronic stress depending on specimen type.	- Affected by time of day when collected (except hair). - - Interpretation challenges since influenced by timing, chronicity, and context.	- Useful in measuring an individual's baseline level prior to experiencing a stressor and the magnitude and duration of an acute response after exposure to a stressor. - Used to assess short-term changes at particular points in the day such as the awakening response or diurnal slope. - Can be analyzed longitudinally at multiple time points (e.g., with saliva or blood) or with 1 to 3 cm of hair to assess enduring or chronic stress.
	DHEA/DHEA-S	- Useful in measuring effective stress regulation and resilience. - Has a longer half-life (DHEA-S) and less diurnal variation than cortisol.	- Interpretation challenges due to significant individual-level, sex and gender variations. - Consideration of confounds such as obesity and BMI needed since amount of adipose tissue affects metabolic clearance rate.	- Useful in measuring an individual's baseline level prior to experiencing a stressor and the magnitude and duration of an acute response after exposure to a stressor (especially DHEA). - Valuable in assessing chronic, sustained levels of the hormone when using hair specimens (DHEA-S a more optimal measure than DHEA of overall baseline levels and hormonal status related to chronic stress).
ANS	Alpha-Amylase	- Useful to assess either basal state or stress reactivity. - Is a “real-time,” nearly instantaneous indicator of acute response to stress with an equally rapid recovery.	- High inter-individual variability (e.g., significantly influenced by age, sex, diet, smoking). - Concentration dependent on how much saliva is produced.	- Useful in measuring an individual's baseline level prior to experiencing a stressor and the magnitude and duration of an acute response after exposure to a stressor. - Less effective as a measure of chronic stress unless paired with cortisol to assess the AA/cortisol ratio over time.
	Heart Rate Variability	- Non-invasive and cost-effective. - Reflects dynamic shifts in ANS regulation, particularly parasympathetic activity. - Sensitive to acute stress and recovery processes.	- Impacted by variability in measurement protocols. - Influenced by respiration, posture, physical activity, medications, and circadian factors.	- Useful in measuring an individual's baseline level prior to experiencing a stressor and the magnitude and duration of an acute response after exposure to a stressor. - Effective for ongoing real time or later assessment of longitudinal activation to varied stressors or the persistence of chronic stress (using wearable sensors).
Immune	Cytokines/CRP	- Direct indicators of inflammatory signals. - Provide insight into immune system activation and systemic inflammation.	- Interpretation and reproducibility influenced by temporal variability of cytokine levels. - Influenced by infection, sleep, medications, adiposity, and other health concerns.	- Useful in measuring an individual's baseline level prior to experiencing a stressor and the magnitude and duration of an acute response after exposure to a stressor (primarily pro-inflammatory cytokines). - Also used in collection of repeated samples over a period of time to monitor change or assess chronic stress (high sensitivity-CRP is the most reliable).
Genetic/epigenetic	Telomeres	- Reflects replicative cellular aging through progressive telomere shortening with cell division. - Captures cumulative effects of chronic stress through pathways involving cortisol, oxidative stress, and inflammation.	- Not sensitive to acute stress or short-term changes. - High inter-individual variability (e.g., influenced by age, genetics, early life stress, and environmental/lifestyle factors).	- Appropriate for measurement of persistent or chronic stress rather than rapid, short-term changes in response to acute stressors.
	DNA Methylation	- Captures the biological embedding of cumulative stress rather than transient responses. - Provides useful insight into intergenerational stress effects. - Has high potential for reversibility.	- Influenced by type of tissue and cells. - Less ideal for measuring immediate responses to acute stress. - Susceptible to confounding from lifetime exposures, making it difficult to isolate the independent effect of stress.	- Primarily used to assess cumulative stress or chronic, longer-term exposure to stressors. - Has shown rapid, short-term shifts in response to acute stressors, suggesting the epigenome has the potential for immediate dynamic flexibility.
Integrated	Multi-Omics Profiles	- Captures coordinated multisystem stress biology. - Can detect subtle biological changes related to stress.	- High cost - Requires advanced analytic techniques. - Interpretation challenges due to multiple interacting signals.	- Captures short-term molecular shifts, such as immediate gene transcription and mRNA fluctuations, as well as immediate metabolic and signaling pathway changes in response to acute stress. - Can be used to evaluate long-term genomic, epigenetic, microbial, and mitochondrial modifications tied to chronic, sustained stressors.

HPA axis, hypothalamic–pituitary–adrenal axis; DHEA, dehydroepiandrosterone; DHEA-S, dehydroepiandrosterone sulfate; ANS, autonomic nervous system; AA, alpha-amylase; CRP, C-reactive protein; mRNA, messenger ribonucleic acid.

**Figure 2:**
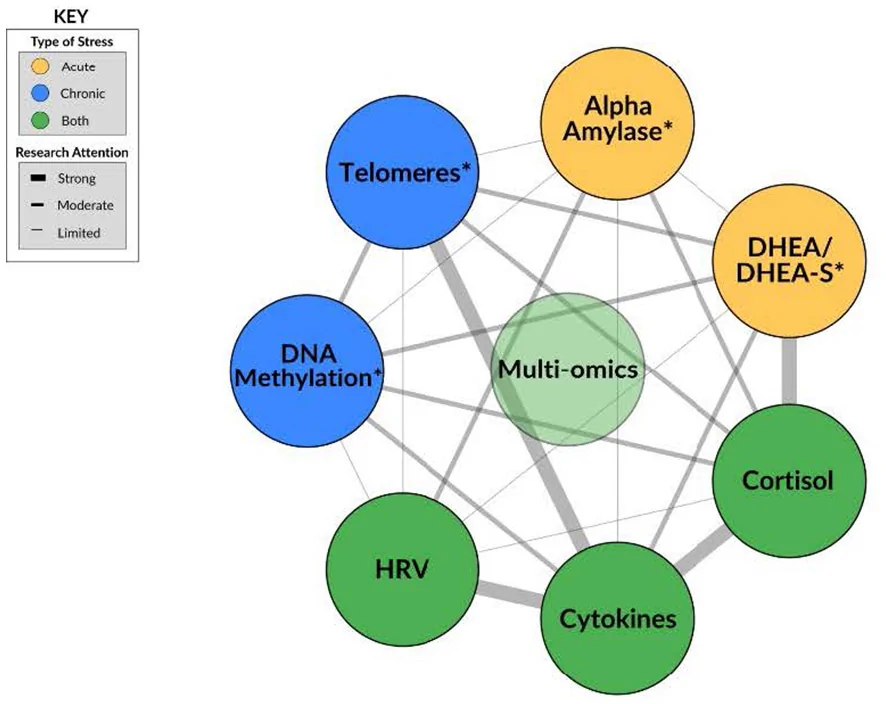
The primary use of each stress biomarker in measurement of acute vs. chronic stress, along with the degree to which linkages between stress biomarkers have been studied. *While colors indicate the *primary* use of these biomarkers in measuring acute or chronic stress, they may be suitable for assessing either type of stress depending on the definition of “acute” or “chronic” and the nature of the research design.

Feasibility of each biomarker's acceptance or use within a particular population and its developmental or cultural context is also important (Gooding et al., 2025; Pra et al., 2024). For example, measures of stress resilience may be more acceptable in light of a community's ethos than measures of stress dysregulation. Or markers using biometric sensors rather than bodily fluids may be more practical when working with a particular age group or setting. Key advantages and limitations of each biomarker are noted in Table 1.

As noted across discussion of biomarkers, it is critical that collection, storage, and processing of biospecimens or sensor-derived, physiologic data employ well-established, validated methods and protocols. Table 2 provides a summary of specimen sources, collection methods, and analytic techniques for each of the biomarkers discussed in this paper. Factors such as incorrect temperature or improper stabilizing agents, contamination, labeling errors, and measurement artifacts (to name a few) can ultimately compromise study results, leading to unreliable and erroneous conclusions. Quality training of research personnel is essential to assure integrity of the data, including detailed instructions related to procedures and their rationale, fidelity and reliability testing for knowledge and adherence to protocols, and certification in biosafety and regulatory compliance.

**Table 2:** Collection and analytical approaches for various stress biomarkers.

System	Biomarker	Specimen sources	Sampling methods	Analytic techniques
HPA axis	Cortisol	Saliva, hair, urine, skin, blood	Repeated timed sampling to assess diurnal rhythm or stress reactivity; single hair collection for chronic stress.	LC-MS/MS, immunoassays (ELISA, chemiluminescent immunoassays).
	DHEA/DHEA-S	Saliva, urine, hair, blood	Standardized timed sampling because of circadian variation; hair collection for chronic hormone assessment.	LC-MS/MS, immunoassays (ELISA, RIA); cortisol/DHEA-S ratio may be calculated as a derived index.
ANS	Alpha-amylase	Saliva	Repeated saliva sampling before, during, and/or after stress exposure or across the day.	Enzymatic colorimetric assays, spectrophotometry; alpha-amylase/cortisol ratio may be calculated as a derived index.
	Heart rate variability	ECG, PPG, wearable biosensors	Continuous physiological recording during resting conditions or standardized stressor paradigms.	Time-domain (RMSSD, SDNN, pNN50) and frequency-domain (FFT, wavelet, LF, HF, LF/HF) analyses of ECG- or PPG-derived HRV signals.
Immune	Cytokines/CRP	Saliva, DBS, blood	Single or repeated biospecimen collection depending on assessment of acute or chronic inflammatory responses.	ELISA, multiplex bead-based immunoassays (e.g., Luminex), high-sensitivity CRP immunoassays.
Genetic/epigenetic	Telomeres	Saliva, buccal, DBS, blood	Cross-sectional or longitudinal DNA sampling; repeated collections when assessing telomere length changes over time.	qPCR, TRF (Southern blot), FISH, STELA, long-read sequencing.
	DNA methylation	Saliva, buccal, hair, blood	Cross-sectional or longitudinal DNA sampling using standardized collection protocols appropriate for the tissue and study design.	Bisulfite conversion, methylation-specific PCR, pyrosequencing, BeadChip arrays, whole-genome bisulfite sequencing.
Integrated	Multi-omics profiles	Saliva, urine, fecal, blood	Coordinated collection of multiple biospecimens according to study objectives and selected-omics platforms.	High-throughput sequencing, mass spectrometry, bioinformatic integration of multi-omics datasets.

HPA axis, hypothalamic–pituitary–adrenal axis; LC-MS/MS, liquid chromatography–tandem mass spectrometry; ELISA, enzyme-linked immunosorbent assay; DHEA, dehydroepiandrosterone; DHEA-S, dehydroepiandrosterone sulfate; RIA, radioimmunoassay; ANS, autonomic nervous system; ECG, electrocardiogram; PPG, photoplethysmography; RMSSD, root mean square of successive differences; SDNN, standard deviation of NN intervals; pNN50, percentage of NN intervals differing by >50 ms; FFT, fast Fourier transform; LF, low frequency; HF, high frequency; HRV, heart rate variability; CRP, C-reactive protein; DBS, dried blood spot; qPCR, quantitative polymerase chain reaction; TRF, terminal restriction fragment; FISH, fluorescence *in situ* hybridization; STELA, single telomere length analysis; PCR, polymerase chain reaction.

Although controlling for potential confounds is necessary in all research, such controls are particularly important with stress-related biomarkers. Because of the circadian rhythms that modulate many of these biomarkers, time of day, sleep-wake cycle, or seasonal variation can influence their basal level or response to stressors. Collecting additional lifestyle data in stress-related biomarker studies, such as information relevant to sleep behaviors and quality, can improve study rigor by accounting for these contextual factors (Sukor et al., 2025; Xia et al., 2021). Differences in normative levels or physiologic reactivity of a marker based on age or sex of the participant are also important considerations (Novais et al., 2016; Volarić et al., 2024). The stress response is influenced by an interplay of biological and psychosocial factors that differ according to sex and gender as well as chronological and developmental age; therefore, both should be considered during analysis and/or interpretation of most stress biomarkers. Further, the number and severity of actual stressful events in participants' lives, which can be affected by multiple sociodemographic factors (e.g., income, occupation), can alter biomarker status (e.g., Hillmann et al., 2024; Pantell et al., 2024; Volarić et al., 2024), contributing to variance in the marker that may be mistakenly attributed to the exposure or intervention being studied. Concurrent health conditions with known alterations in neuroendocrine, immunologic, and/or genomic systems (e.g., obesity, cancer) must also be adjusted for during analysis and interpretation of findings. In addition to these salient confounds that have broad relevance across biomarkers, there is an additional need to control for factors specific to medical treatments of study participants that may modify individual markers (e.g., treatment of allergies or arthritis with corticosteroids when measuring cortisol levels). Medical history information such as diagnoses and treatments, and other health information such as body mass index, are important data to gather as additional context for the study of stress biomarkers.

The use of one stress-related biomarker will provide valuable but limited information about the stress of an individual or population. Integrating this data with other stress-related information will provide a more comprehensive understanding of stress and its significance. Additional data can include (a) the individual's feelings and thoughts about their stress experience and coping ability, and (b) actual stressors which they encounter, along with duration and gravity of the stressors. Biomarkers of stress from the same or different stress-response systems often collaborate with one another to regulate stress or are influenced concurrently during exposure to stressors. This interplay is an essential feature of multi-omics. However, it is also a salient consideration among the individual markers we have discussed. The conceptual model shown in Figure 3 underscores the nuanced interactions and integrative nature of the biological networks involved in catalyzing or managing stress. Thus, examining two or more stress biomarkers will yield a more comprehensive understanding of the mechanisms involved in any effects of stress or the stress response. The graph in Figure 2 shows the amount of research attention that has been given to examining the relationships between various biomarkers to date. Some relationships have been studied quite extensively (e.g., cortisol-DHEA; telomeres-cytokines) while other relationships have received minimal attention (e.g., DNA methylation-heart rate variability). Clearly, more knowledge is needed to fully understand the ways in which biomarkers may catalyze, mediate, or moderate one another's effects or act synergistically in response to stress.

**Figure 3:**
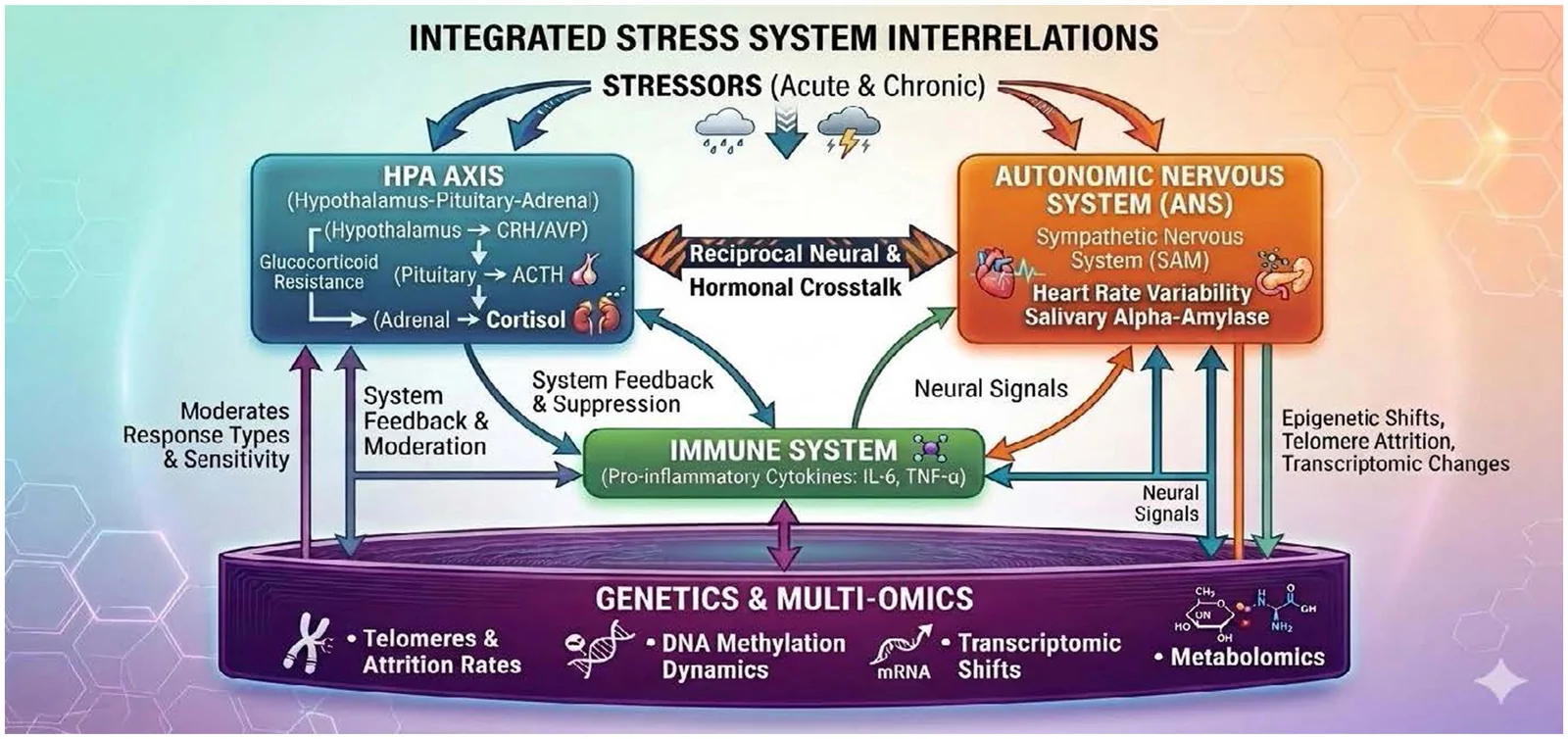
Relationships among biological systems associated with the stress response.

## Future research on biomarkers of stress

Although the field can benefit from more research on stress biomarkers overall, we would like to mention a few key areas needing special attention. One area is the identification of reference ranges that are considered optimal or normative vs. suboptimal or dysregulated for the various stress biomarkers. These are not currently available for many biomarkers used in stress-related research. Understanding normative vs. atypical values of these biomarkers will also necessitate research to distinguish ranges that may apply in states of acute vs. chronic stress. Research has shown for markers such as cortisol, HRV, and C-reactive protein that basal values are significantly different among individuals who have endured persistent or chronic exposure to stressors than among those who have not. However, these distinctions need more in-depth research and have not been well-studied for most stress biomarkers. The lack of reference ranges specific to different stages of the lifespan is a particularly critical research gap, often limiting interpretation of findings specific to infancy and childhood/adolescence as well as trajectories across adulthood from young to old age. A second research need is the identification of specific sociodemographic factors that should be consistently included as covariates in all stress-related biomarker research. For instance, a number of factors have been frequently associated with baseline stress levels and biological responses to stress but have not been examined for validity and reliability across all biomarkers. Examples include age, biological sex, race and ethnicity, and socioeconomic status. Data to support these and other crucial covariates can then be universally recommended for inclusion in statistical models. Third, increased research on circadian rhythms is essential to clarify their effects on each biomarker. Although more research exists on rhythms of some stress biomarkers than others (e.g., cortisol, cytokines), circadian effects have not been carefully studied; and the majority of studies do not recognize or control for these potential effects in their designs. The lack of such controls can lead to an inability to compare or integrate data across studies, as we attempt to develop knowledge in the field. Fourth, comparison of different assay methods and their results is critical. Limited research which has examined these differences for certain biomarkers indicates that different methods can produce different findings and that some methods are clearly superior to others. Augmented efforts to conduct such studies are essential to assure valid and comparable findings across studies.

More research is also needed on the effectiveness of various stress tests or stressor paradigms that are used in research to examine stress reactivity and regulation. Paradigms such as the “Trier Social Stress Test” are often considered a gold standard for many types of research (Labuschagne et al., 2019; Narvaez Linares et al., 2020). However, there is a need to identify tests that are most appropriate for specific developmental stages, sociodemographic populations, and health problems. Different stressor paradigms activate different biological pathways and will have variable relevance to some health problems or stress biomarkers. In addition, some paradigms require culture or age-specific language and idioms, physical exertion, or induced cognitive overload which could be biased, invalid, unsafe, or unethical in certain populations or illness groups. Increased focus on the development and testing of stressor paradigms will enhance our ability to trust data that are derived from exposure to specific stress tests and the comparability of findings when stressor paradigms are used in various studies. Sixth, studies are increasingly examining differences in the results they find when using various types of biospecimens (e.g., blood, saliva, urine, hair) as sources for a biomarker. Investigators working with certain biomarkers have given more attention to these comparisons than others. Research shows that different specimen types can influence the quantity and quality of the data, with some being more optimal than others for different research aims or settings and different results being produced across specimens under similar conditions. Evaluation of different specimen types should be increased and encouraged for all stress-related, biochemical markers. Similarly, further comparison of physiological, biometric technologies for HRV measurement is needed (e.g., ECG vs. PPG). Next, a greater emphasis on resilience to stressors is essential in all stress-related biomarker research. Most research to date has focused on the relationships of biomarkers to disease or illness, including characteristics of the markers that may reflect risk for illness or the actual diagnosis/presence of an illness. While some of the biomarkers we discuss here are recognized as valuable measures when examining resilience to stress (e.g., DHEA, HRV), most warrant increased attention to characteristics of the marker that indicate an ability to adapt well in the face of significant stress and/or return to a state of wellbeing after major adversity, trauma or crisis.

Another area for further study encompasses an array of important and emerging biomarkers that are not addressed in this paper. BDNF (Brain-Derived Neurotrophic Factor) is a protein that promotes the growth, survival, and maintenance of neurons. Studies have shown increases of BDNF in short term stress and reductions in chronic stress (Lima Giacobbo et al., 2019; Linz et al., 2019). Perturbations in BDNF levels and DNA methylation associated with these different types of stress warrant attention. Secondly, melatonin (an amine hormone) and oxytocin (a peptide hormone) both have primary functions unrelated to stress; however, they also are both considered counter-regulators of the HPA axis, dampening and inhibiting the body's primary stress response (Onaolapo et al., 2017; Takayanagi and Onaka, 2021). As such, a greater understanding of their role in stress reactivity and recovery is needed. A third stress marker of research interest, related to our focus on “multi-omics,” involves extracellular vesicles (EVs). These are tiny fluid-filled packages released by stressed cells that carry “cargo” such as miRNAs, mRNA, DNA, proteins, and metabolites to other cells to signal threat or help the body adapt to stressors (Makrygianni and Chrousos, 2023). EVs are proposed to have an important role in restoring homeostasis during acute stress (Crewe, 2023) and in managing chronic stress (Sakamoto et al., 2021). However, the specific mechanisms underlying these processes need further study.

Because of their proliferation and potential value to science, the utility of biosensors in stress assessment and monitoring is another vital area for research. Earlier in the paper, we discussed the importance of sensors for measurement of heart rate variability; but sensors have relevance for other biomarkers as well. For example, they are used in the study of skin electrical conductance (electrodermal), catecholamine and cortisol levels in sweat, and skin temperature changes caused by blood vessel constriction or dilation under stress. Although wearable sensors enhance the ability to collect stress-related data in real time, research needs to address some key challenges associated with their use. Real-world movement and other environmental exposures introduce extensive noise and false signals into the data being collected via sensors, requiring development of robust machine learning models to accurately filter out artifacts (Tarasov et al., 2026). Research needs to identify how to cleanly distinguish sensor-derived data associated with true psychological stress from data caused by physical exertion or other endogenous and exogenous stimuli. Artificial intelligence and machine learning have special relevance in addressing this challenge; but they also have broader implications for stress-related biomarker research. Supervised and unsupervised training approaches can be used to predict or classify patterns of biological response within or across systems using vast amounts of biological data from varied sources (Al Abdi et al., 2026; Onim et al., 2024). These approaches may ultimately identify biosignatures of stress sensitivity and response that are not recognized by more traditional methods (Alowais et al., 2023). Machine-learning models, such as support-vector methods, random forest models, and Bayesian networks, have been able to predict self-reported stress with high accuracy when using a set of biological variables (Mentis et al., 2024; Vaessen et al., 2021).

Lastly, the salience of stress biomarkers to precision health should be noted (Le-Niculescu et al., 2020), along with the need for research that can advance more targeted, individualized stress assessment and treatment through biomarkers. Integrating data from biomarkers provides the opportunity to identify unique phenotypes of stress vulnerability and stress response through examining interrelationships among molecular characteristics, physiological and chemical states, and cellular features as they shape specific stress profiles. In addition to the identification of stress phenotypes that may indicate distinct profiles within a group or population, the integration of multiple biomarkers can also be used for personalized mapping of a particular individual's stress response, either in real time or through examination of their data across time and data sources. However, many challenges face the investigator when attempting to integrate data from multiple biomarkers, including data heterogeneity, cross-platform synchronization, and the need for robust validation of machine learning algorithms to prevent misinterpretation. Developing methods to effectively normalize and synthesize data across different platforms is an area of particular research need. Ethical issues surrounding data privacy and the transparency of informed consent are also essential areas for research when using methods such as multi-omics and smartphone tracking or other digital phenotyping. Still, complex analysis of multiple biochemical and sensor-derived data is timely and possible through available bioinformatics platforms, using multivariate and time-series statistical methods as well as machine learning models. Ultimately, research in these areas can enable prediction, assessment, and prevention of stress-related mental and physical illness as well as the creation of precision health profiles that permit tailoring of interventions to an individual's specific stress biosignature.

In essence, the biological component of stress makes a unique contribution to understanding the stress experience, providing information that is distinct from stress-related feelings and thoughts. As a result, biomarkers represent a critical asset in the overall scope of research on stress and stress regulation, advancing knowledge of their health-related impact in fundamental and significant ways.
